# Assessing robustness of radiomic features by image perturbation

**DOI:** 10.1038/s41598-018-36938-4

**Published:** 2019-01-24

**Authors:** Alex Zwanenburg, Stefan Leger, Linda Agolli, Karoline Pilz, Esther G. C. Troost, Christian Richter, Steffen Löck

**Affiliations:** 1 OncoRay – National Center for Radiation Research in Oncology, Faculty of Medicine and University Hospital Carl Gustav Carus, Technische Universität Dresden, Helmholtz-Zentrum Dresden - Rossendorf, Dresden, Germany; 2 National Center for Tumor Diseases (NCT), Partner Site Dresden, Dresden, Germany; 3 German Cancer Consortium (DKTK), Partner Site Dresden, and German Cancer Research Center (DKFZ), Heidelberg, Germany; 4 Department of Radiotherapy and Radiation Oncology, Faculty of Medicine and University Hospital Carl Gustav Carus, Technische Universität Dresden, Dresden, Germany; 5 Helmholtz-Zentrum Dresden - Rossendorf, Institute of Radiooncology – OncoRay, Dresden, Germany

## Abstract

Image features need to be robust against differences in positioning, acquisition and segmentation to ensure reproducibility. Radiomic models that only include robust features can be used to analyse new images, whereas models with non-robust features may fail to predict the outcome of interest accurately. Test-retest imaging is recommended to assess robustness, but may not be available for the phenotype of interest. We therefore investigated 18 combinations of image perturbations to determine feature robustness, based on noise addition (N), translation (T), rotation (R), volume growth/shrinkage (V) and supervoxel-based contour randomisation (C). Test-retest and perturbation robustness were compared for combined total of 4032 morphological, statistical and texture features that were computed from the gross tumour volume in two cohorts with computed tomography imaging: I) 31 non-small-cell lung cancer (NSCLC) patients; II): 19 head-and-neck squamous cell carcinoma (HNSCC) patients. Robustness was determined using the 95% confidence interval (CI) of the intraclass correlation coefficient (1, 1). Features with CI ≥ 0:90 were considered robust. The NTCV, TCV, RNCV and RCV perturbation chain produced similar results and identified the fewest false positive robust features (NSCLC: 0.2–0.9%; HNSCC: 1.7–1.9%). Thus, these perturbation chains may be used as an alternative to test-retest imaging to assess feature robustness.

## Introduction

Radiomics is the high-throughput quantitative analysis of medical imaging to facilitate model-based treatment decisions^1,2^. A prevalent approach relies on the computation of image biomarkers (features) within a region of interest (ROI). In this approach features quantify different aspects of the ROI, such as mean intensity, volume and texture heterogeneity. Variations in patient positioning, image acquisition and segmentation affect each feature to varying degrees^3,4^. If radiomic models use features that are not robust against such influences, they will perform poorly when applied to new data^5^. Assessing feature robustness is thus recommended to improve generalisability of radiomic models.

Non-robust image features are commonly identified using test-retest imaging^6–10^. In test-retest imaging, the same region of interest is imaged twice within a time interval of minutes to days, usually with the same acquisition protocol. Consequently, these two images are similar, but not identical, which allows the identification of non-robust features. After identification, non-robust features are excluded from further analysis.

Although the identification of robust features is important, implementing test-retest imaging for every radiomic study has been difficult to achieve for several reasons. First, feature robustness is dependent on the phenotype of interest as well as the imaging modality. This means that information concerning feature robustness cannot be transferred between studies on different phenotypes^11^ and modalities^7^. Furthermore, feature values depend on multiple factors, including the voxel size and discretisation used^12–14^. Thus, even if a previous study determined feature robustness for a particular phenotype and modality, the results may not be transferable due to the use of different computational settings. Second, test-retest imaging may be difficult to obtain generally, as it is not part of the clinical routine. Acquiring test-retest imaging would thus require additional resources in terms of personnel and imaging time, and, potentially, an increased patient radiation dose. An alternative would be to use the appropriate publicly available test-retest data set, but such data are likewise sparse.

It would therefore be convenient if feature robustness against perturbations could be assessed from single images. To do so, we can use methods more prevalent in the deep learning computer vision field. Here, networks are constructed to be invariant to various perturbations, e.g. noise, rotation and translation^15^. To achieve invariance, such perturbations are created on purpose, distorted images are generated and subsequently used as input data to develop deep learning models. The same principle may apply to the hand-crafted features that are considered in this work. We hypothesise that perturbations of single images may successfully identify the majority of features that are not robust in test-retest imaging. The aim is thus to identify perturbations that minimise the number of false positive robust features, using robustness in test-retest imaging as reference.

## Results

Two test-retest data sets of computed tomography (CT) images were assessed, namely: (I) a publicly available non-small cell lung cancer (NSCLC) cohort of 31 patients; and (II) an in-house head and neck squamous cell carcinoma (HNSCC) cohort of 19 patients.

**Figure 1:**
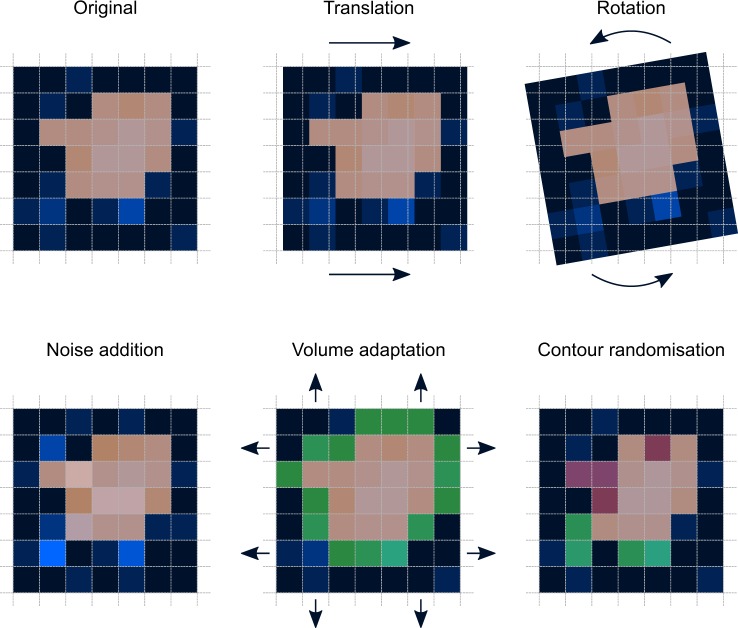
Perturbation examples. To perturb an image (blue) and the region of interest mask (orange overlay), the original image is translated, rotated, noised, and has its mask adapted and randomised. Translation and rotation change both the image and its mask, whereas noise only distorts the image. Volume adaptation and contour randomisation change the mask by adding (green overlay) and removing voxels (red overlay). Note that translation and rotation require additional interpolation (not shown).

After delineating the gross tumour volume (GTV), the CT images were perturbed by rotation (R), Gaussian noise addition (N), translation (T), volume adaptation (growth/shrinkage of the ROI mask; V) and supervoxel-based contour randomisation (C), see Fig. 1 and Table 1. Eighteen combinations of perturbations were created by chaining perturbation operations. All chains involved repetition with different settings or randomisation. Morphological, statistical and texture features (4032 in total) were computed from the GTV ROI in each distorted image.

**Table 1:** List of perturbations, with their abbreviation and the number of different images generated by each perturbation.

perturbation	abbreviation	number of perturbed images
rotation	R	27
noise addition	N	30
translation	T	27
volume adaptation	V	29
contour randomisation	C	30
rotation and translation	RT	32
rotation, noise addition and translation	RNT	32
rotation and volume adaptation	RV	30
rotation and contour randomisation	RC	27
translation and volume adaptation	TV	40
translation and contour randomisation	TC	27
rotation, translation and contour randomisation	RTC	32
rotation, noise addition, translation and contour randomisation	RNTC	32
volume adaptation and contour randomisation	VC	30
rotation, volume adaptation and contour randomisation	RVC	30
rotation, noise addition, volume adaptation and contour randomisation	RNVC	30
translation, volume adaptation and contour randomisation	TVC	40
noise addition, translation, volume adaptation and contour randomisation	NTVC	40

The settings used by each perturbation chain are listed in Supplementary Note 5.

**Figure 2:**
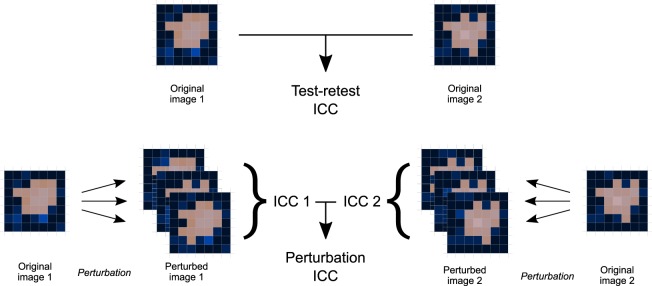
Workflow to determine the test-retest and perturbation intraclass correlation coefficients (ICC) for each feature. The test-retest ICC was calculated directly between the same features in both images. To derive the perturbation ICC, an ICC was first calculated between feature values in perturbations of image 1 (ICC 1) and then again in perturbations of image 2 (ICC 2). The perturbation ICC is the average of ICC 1 and 2.

Robustness of each feature was measured by the intraclass correlation coefficient (1, 1) (ICC)^16^. We computed the ICC of a feature between either the test and retest images (test-retest ICC), or between the perturbed images of each perturbation chain (perturbation ICC), see Fig. 2. The 95% confidence interval (CI) of the ICC was then used to determine robustness by comparing with a threshold of 0.90^17^. Thus, a feature is robust if CI ≥ 0.90, non-robust if CI < 0.90, and has an indeterminate robustness if the CI overlaps with the threshold.

A table containing all estimated ICC values and their 95% confidence intervals for all features and both cohorts was appended as supplementary data.

### Comparison between NSCLC and HNSCC cohorts

To validate the basic premise that feature robustness is dependent on the phenotype, we compared feature robustness based on the test-retest ICC in both cohorts.

In the NSCLC cohort 2310 (57.3%) features were found to be robust, 597 (14.8%) were non-robust and 1125 (27.9%) had an indeterminate robustness. In the HNSCC cohort 582 (14.4%) features were robust, 1369 (34.0%) were non-robust and 2081 (51.6%) had an indeterminate robustness.

454 (11.3%) and 280 (6.9%) features were robust and non-robust in both cohorts, respectively. Additionally, 656 (16.3%) features were robust in the NSCLC cohort, but not in the HNSCC cohort, and 35 (0.9%) features were robust in the HNSCC cohort, but not in the NSCLC cohort. The remainder could not be compared due to indeterminate robustness in the NSCLC cohort (526; 13.0%), the HNSCC cohort (1482; 36.8%) or both cohorts (599; 14.9%).

### Robustness under image perturbations

**Figure 3:**
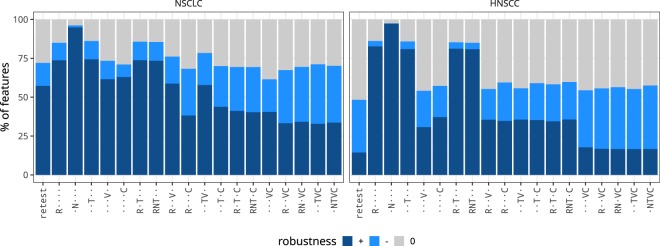
Overall robustness of features for test-retest and perturbation conditions. Robustness was determined using the 95% confidence interval (CI) of the intraclass correlation coefficient. Features with CI ≥ 0.90 were considered to be robust (+), CI < 0.90 non-robust (−), and indeterminate (0) otherwise. Perturbations are abbreviated, see Table 1: R: rotation; N: noise addition; T: translation; V: volume adaptation; C: contour randomisation.

The fraction of robust features for test-retest imaging and image perturbations is shown in Fig. 3. In both cohorts, the N perturbation yielded the highest number of robust features (NSCLC: 95.0%; HNSCC: 97.4%), which was higher than the number of robust features as determined by test-retest imaging (NSCLC: 57.3%; HNSCC: 14.4%). The lowest number of robust features in the NSCLC cohort was identified by the TVC perturbation chain (32.9%), followed by RVC (33.3%), NTVC (33.7%), RNVC (34.2%) and RC (38.3%). In the HNSCC cohort, TVC (16.6%), NTVC and RNVC (both 16.7%), RVC (16.8%), VC (17.8%) and V (30.8%) identified fewest robust features.

### Feature-wise comparison of perturbation and test-retest robustness

**Figure 4:**
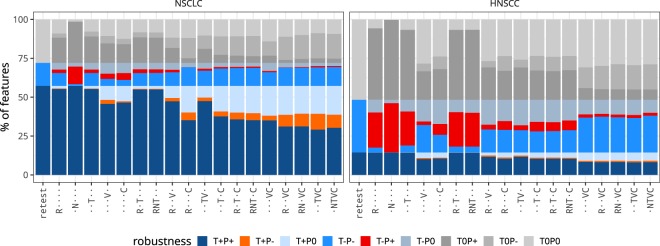
Feature-wise comparison of robustness under test-retest and perturbation conditions. Robustness was determined using the 95% confidence interval (CI) of the intraclass correlation coefficient. Features with CI ≥ 0.90 were considered to be robust (+), CI < 0.90 non-robust (−), and indeterminate (0) otherwise. By comparing robustness states between test-retest (T) and perturbation (P) conditions, a feature was either robust under both conditions (T+P+; true positive), non-robust under both conditions (T−P−; true negative), only robust under perturbations (T−P+; false positive), or only robust under test-retest conditions (T+P−; false negative). The state of the remaining features is either indeterminate due to overlap of the test-retest CI with the threshold (T0P−, T0P+), overlap of the perturbation CI with the threshold (T + P0, T − P0) or both (T0P0). Test-retest robustness was used as reference, and the corresponding column therefore only contains true positives and negatives, as well as indeterminate robustness. Perturbations are abbreviated, see Table 1: R: rotation; N: noise addition; T: translation; V: volume adaptation; C: contour randomisation.

Test-retest and perturbation robustness were also compared directly for the same feature. Thus, when comparing test-retest and perturbation robustness for each feature, a feature may be robust under both perturbation and test-retest conditions, non-robust under both, robust under test-retest or perturbation conditions only, or of indeterminate robustness. Using test-retest robustness as a reference, these conditions represent true positive, true negative, false negative, false positive and indeterminate cases, respectively. The direct feature-wise comparison of robustness is presented in Fig. 4.

No perturbation identified every feature that was non-robust under test-retest conditions in both cohorts. The number of false positives differed between perturbations and cohorts. Perturbation chains in the NSCLC cohort yielded less false positives than the HNSCC cohort on average (2.0% vs. 9.4%).

In the NSCLC cohort, the RC perturbation chain caused the lowest number of false positives (0.0%), followed by RVC (0.2%), RNVC (0.5%) and NTVC (0.7%). The lowest false positive fraction in the HNSCC cohort was produced by RNVC perturbation chain (1.7%), followed by RVC (1.8%), TVC and NTVC (both 1.9%). In the HNSCC cohort, the RC perturbation chain led to 5.7% false positives.

## Discussion

We compared several methods for perturbing images to determine feature robustness. The perturbation chains that combine rotation or translation with volume adaptation and contour randomisation (RVC, RNVC, TVC, NTVC) led to a low number of false positives in both cohorts, using test-retest robustness as reference, and where otherwise comparable. Hence any of these chains may be used as an alternative to test-retest imaging to assess feature robustness.

Other perturbation methods performed poorly, particularly if only one kind of perturbation was used. This includes methods such as noise addition or simple rotations or translations. The combination of rotation and translation was not better than rotation or translation alone. Chaining perturbation methods that primarily alter the intensity content (noise, translation, rotation) with methods that update the region of interest mask (volume adaptation and contour randomisation) improved results in terms of less false positives with regard to test-retest imaging.

Considerable difference in overall robustness was observed between NSCLC and HNSCC cohorts. Specific image processing parameters or contributions of particular feature family are unlikely to cause this difference (Supplementary Notes 7 and 8). The differences are more likely caused by either inherent differences between tumour phenotypes^11^ or by limitations inherent to test-retest imaging in patients. As only two test-retest images are usually acquired in patients, the number of possible acquisition options that can be assessed is constrained. Lack of access to raw imaging data to assess different reconstruction settings compounds this limitation. In this study, two different image acquisition and reconstruction protocols were used in the HNSCC cohort, whereas only one protocol was used for test-retest imaging in the NSCLC cohort. In the HNSCC cohort exposure and reconstruction kernels differed between protocols (Supplementary Note 1). The exposure between both HNSCC images differed by a factor 4 on average, whereas exposure in the NSCLC set was similar between images. The HNSCC test-retest set may thus have captured differences in exposure. However, the effect of exposure and tube current on feature robustness has been contested. Larue *et al*. and Mackin *et al*. both found that exposure had a marginal effect on feature robustness^18,19^, whereas Midya *et al*. found that it had a more pronounced effect^20^. The HNSCC test-retest set may also have been affected by the difference in reconstruction kernels. Though both kernels in the HNSCC cohort produce smooth images, differences in reconstruction kernels may strongly affect feature values^21,22^.

Aside from the overall difference in robustness between the NSCLC and HNSCC cohorts, a large difference in indeterminate robustness fractions can be observed between both cohorts. This is reflected in the 95% confidence interval of the ICC value of each feature. The average width of the 95% confidence interval of test-retest ICCs was 0.12 (NSCLC) and 0.35 (HNSCC). This indicates that feature values in the HNSCC cohort were less consistent between both images of the test-retest set, which may be related to the aforementioned difference in acquisition and reconstruction protocols. Yet, the decreased consistency between test and retest images may also be related to delineation uncertainties. The potential role of delineation uncertainties may observed by comparing the single perturbations for volume adaptation and contour randomisation between both cohorts with perturbations that only affect intensities. In the NSCLC cohort, delineation perturbations affect feature robustness less than in the HNSCC cohort, which was also found by Pavic *et al*.^23^.

Image perturbations allows performing repeated measurements without actual acquisition of multiple images, which could be considered an advantage over test-retest imaging. We consider three methods for incorporating repeated measurements into radiomics modelling. The first, straightforward, method is to include only robust features in the modelling process, and omit indeterminate and non-robust features. This method is commonly used when robustness is determined using test-retest imaging and its implementation into a modelling workflow should therefore be easy^5^. Moreover, this method is useful when only a subset of the development cohort is perturbed, or a separate data set is used for robustness analysis.

It should be noted that the number of indeterminate features correlates with the number of perturbations, as the 95% confidence interval of the ICC shrinks with increasing repeated measurements. It is thus possible to increase the number of robust and non-robust features by increasing the number of perturbations, albeit with diminishing returns. Many studies sidestep this issue entirely by applying a threshold against the estimated ICC^24^ instead of its confidence interval. This criterion is less stringent than comparison against the confidence interval and may lead to the inclusion of features that have reasonable probability (between 2.5 and 50%) of actually not meeting the criterion. This is particularly risky if the confidence intervals are wide and overlap with ICC values < 0.50 (poor robustness) and 0.50 ≥ ICC < 0.75 (moderate robustness)^17^. Thus, if a confidence interval is provided with an ICC value, it would be preferable to use this interval instead of the estimated ICC for selecting robust features.

The second way to use repeated measurements for radiomics modelling is by averaging the measurements for each feature. Averaging suppresses noise and as a consequence the corresponding panel ICC is always higher than that of a single measurement^16^, and its 95% confidence interval smaller. The mean values of the features that are robust according to the panel ICC are then included in the modelling process. This method requires that all images in the development cohort are perturbed, and may thus computationally be more expensive than the first.

The final method builds upon the second, and is conceptually close to the use of image perturbations for deep learning. Instead of averaging values and selecting robust features prior to modelling, all values are included in the model development process. One advantage of this method is that information concerning the distribution of feature values within and across samples is not lost, and may be exploited during the model development process. Another advantage is that an explicit robustness threshold is not required. However, this method does require that all images in the development cohort are perturbed and may add complexity to radiomics modelling frameworks. A future study should compare the three methods and their effect on the performance of radiomic models.

One limitation of the current study is that we only assessed test-retest imaging based on computed tomography, as test-retest data sets for other modalities were not available to us. The proposed methodology should be assessed for other modalities, e.g. positron emission tomography (PET) and magnetic resonance imaging (MRI). Some image perturbation parameters, such as the volume of supervoxels, may require revision for other modalities.

Another limitation of the current study is that we did not assess the effect of expert delineation uncertainties directly. As mentioned before, delineation uncertainties also cause variability in feature values^23^. Volume adaptation and contour randomisation perturbations try to induce this uncertainty, but a comparison against a multiple delineation data set should be performed in the future.

In conclusion, we investigated the use of image perturbations to determine the robustness of radiomic features, using test-retest imaging as reference. Our findings indicate that perturbation methods that distort image intensities and deform the ROI mask (NTVC, TVC, RNVC and RVC) may be used as an alternative to test-retest imaging to determine feature robustness.

## Methods

### Test-retest cohorts

Two patient cohorts with test-retest computed tomography imaging were used: a publicly available non-small cell lung cancer cohort of 31 patients^25,26^ and an in-house cohort (DRKS 00006007) of 19 patients with locally advanced head and neck squamous cell carcinoma^27^. The NSCLC cohort is available from the Cancer Imaging Archive^28^. For the NSCLC cohort, two separate images were acquired within 15 minutes of each other, using the same scanner and acquisition protocol. Images in the HNSCC cohort were acquired within 4 days of each other using a different protocol, i.e. one CT image was acquired for ^18^F-Fludeoxyglucose positron emission tomography (PET) attenuation correction, and the other for attenuation correction of ^18^F-Fluoromisonidazole PET. Image acquisition parameters for both cohorts are shown in Supplementary Note 1.

Informed consent was obtained from all patients. Approval for analysis of the in-house data set was provided by the local ethics committee (Ethikkomission an der TU Dresden: EK 177042017). This study was conducted according to relevant guidelines and regulations.

The GTV was delineated by experienced radio-oncologists (L.A., K.P., E.G.C.T) using the Raystation 4.6 treatment planning system software (RaySearch Laboratories AB, Stockholm, Sweden), and subsequently used as the region of interest.

### Image processing

**Figure 5:**
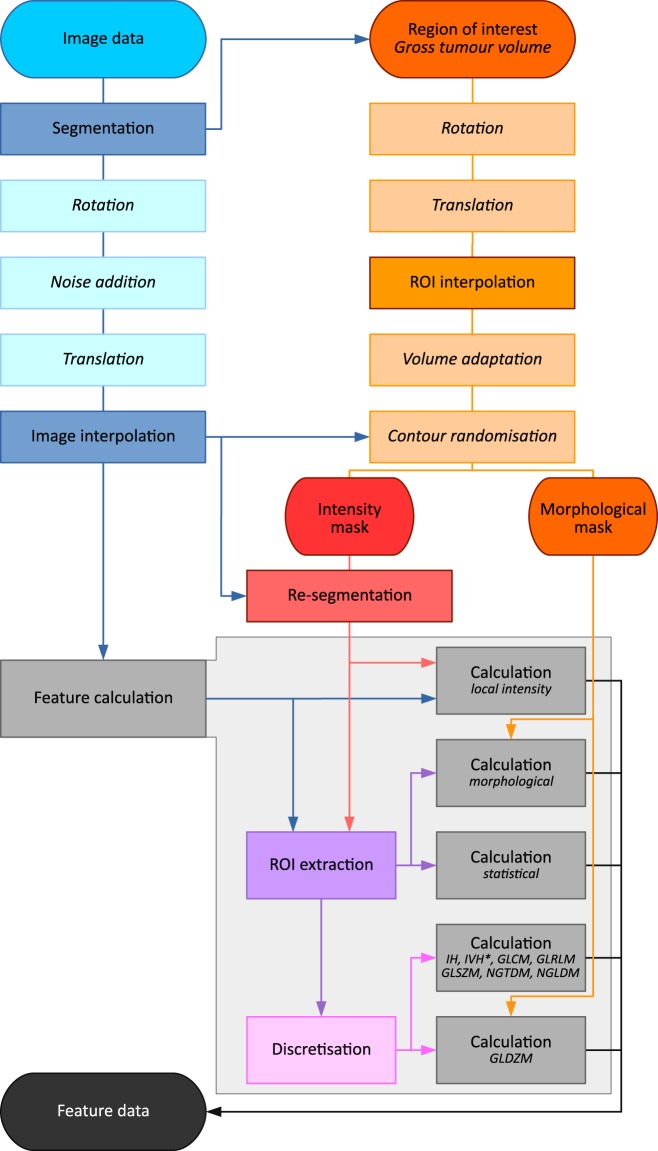
Image processing scheme with perturbations. A computed tomography (CT) image and a segmented gross tumour volume (GTV) are used as the input image data and the region of interest (ROI) respectively. The CT and ROI are processed to compute image features. Rotation, translation, noise addition, volume adaptation and contour randomisation are optional perturbation steps. Other image processing steps are detailed in the documentation of the image biomarker standardisation initiative (IBSI)^29^. IH: intensity histogram; IVH: intensity-volume histogram; GLCM: grey level co-occurrence matrix; GLRLM: grey level run length matrix; GLSZM: grey level size zone matrix; GLDZM: grey level distance zone matrix; NGDTM: neighbourhood grey tone difference matrix; NGLDM: neighbouring grey level dependence matrix. This figure is based on the image processing scheme in the IBSI document.

Image processing was conducted using the scheme and recommendations provided by the Image Biomarker Standardisation Initiative (IBSI)^29^. An overview of the processing steps is provided in Fig. 5, and further details may be found in the IBSI documentation. A complete overview of the image processing parameters, excluding perturbation-related parameters, may be found in Table 2, and are reported in compliance with the preliminary IBSI reporting guidelines^29,30^.

**Table 2:** Image processing parameters for both NSCLC and HNSCC data sets.

parameter	NSCLC	HNSCC
interpolated isotropic voxel spacing (mm)	1, 2, 3, 4	1, 2, 3, 4
pre-interpolation filter	gaussian, *β* = 0.93	gaussian, *β* = 0.93
image interpolation method	trilinear	trilinear
image intensity rounding	to nearest HU	to nearest HU
ROI interpolation method	trilinear	trilinear
ROI mask partial volume threshold	0.5	0.5
re-segmentation range (HU)	[−300, 200]	[−150, 180]
re-segmentation outlier threshold	±3*σ*	±3*σ*
discretisation		
fixed bin number (bins)	8, 16, 32, 64	8, 16, 32, 64
fixed bin size (HU)	6, 12, 18, 24	6, 12, 18, 24

The isotropic voxel spacing is defined in three dimensions, i.e. a spacing of 2 mm corresponds to a voxel dimension of 2 × 2 × 2 mm. Discretisation was performed using two methods (*fixed bin number* and *fixed bin size*) with varying bin sizes. *ROI*: region of interest; *HU*: Hounsfield unit; *σ*: standard deviation of voxel intensities within the region of interest.

In short, after loading a CT image, DICOM RTSTRUCT polygons were used to generate a voxel-based segmentation mask for the GTV ROI. The image and mask were then both rotated over a set angle *θ* (optional). Gaussian noise, based on the noise levels present in the original image, was added to the image (optional). Subsequently, both image and mask were translated with a sub-voxel shift *η* (optional) and interpolated with prior Gaussian anti-aliasing (Supplementary Note 2). After interpolating to isotropic voxel dimensions, the image intensity values were rounded to the nearest integer Hounsfield unit, and the mask was re-labelled based on the partial voxel volume threshold. The mask was then grown or shrunk to alter the volume by a fraction *τ* (optional), before being perturbed by supervoxel-based contour randomisation^31^ (optional). The mask was subsequently copied to generate an intensity mask and a morphological mask. The intensity mask was re-segmented to an intensity range which includes only soft-tissue voxels. Voxels with intensities deviating more than three standard deviations from the mean of the ROI were excluded from the intensity mask as well^32,33^. The image and both masks were subsequently used to compute radiomic features, with several feature families requiring additional discretisation (Supplementary Note 3).

### Image perturbations

Five basic image perturbation methods were implemented in the image processing scheme described above. These were rotation (R), noise addition (N), translation (T), volume adaptation (V) and contour randomisation (C). Examples are shown in Fig. 1. Rotation perturbs the image and mask by performing an affine transformation that rotates the image and mask in the axial (*x*, *y*) plane, i.e. around the *z*-axis, for a specified angle $\theta \in [\,-\,{13}^{\circ },\,{13}^{\circ }]$. Noise addition perturbs image intensities by adding random noise that was drawn from a normal distribution with mean 0 and a standard deviation equal to the estimated standard deviation of the noise present in the image. Translation perturbs the image and mask by performing an affine transformation that shifts the image and mask for specified fractions $(\eta \in \mathrm{[0.00,}\,\mathrm{0.75]})$ of the isotropic voxel spacing along the *x*, *y* and *z* axis. Volume adaptation grows and/or shrinks the mask by a specified fraction $\tau \in [\,-\,\mathrm{0.28,}\,\mathrm{0.28]}$. Contour randomisation is based on simple linear iterative clustering^31^, and perturbs the mask by randomly selecting supervoxels based on the overlap with the original mask. The algorithmic implementation of these perturbations is described in Supplementary Note 4.

Perturbations were chained using the settings documented in Supplementary Note 5. Each rotation angle and volume adaptation fraction led to generation of a new image. Noise addition and contour randomisation could be repeated multiple times, with each repetition producing a new perturbed image as well. The translation fraction was permuted over the different directions. For example, for translation fractions $\eta =\{\mathrm{0.25,}\,0.5\}$, 2^3^ = 8 permutations were generated. Each permutation generated a new image. When chaining perturbations, all provided parameters were permuted.

An overview of the perturbation chains and the number of perturbed images created is shown in Table 1. All perturbation chains produced between 27 and 40 perturbed images.

### Features

All features defined in the IBSI documentation were implemented^29^, leading to a set of 182 base features that were used to assess morphological, statistical and texture characteristics of the ROI. These base features belong to the morphological, local intensity, intensity-based statistical, intensity-histogram, intensity-volume histogram, grey level co-occurrence matrix-based texture, grey level run length matrix-based texture, grey level size zone matrix-based texture, grey level distance zone matrix-based texture, neighbourhood grey tone difference matrix-based texture, and neighbouring grey level dependence matrix-based texture feature families. All base features were computed at multiple scales, namely for isotropic voxel spacings of 1, 2, 3 and 4 mm^34^. 118 base features required discretisation. Both fixed bin size and fixed bin width discretisation algorithms were used, each with four settings. Thus, a total of 4032 features were computed in each image. Supplementary Note 3 contains further details with regard to feature computation.

Both image processing and feature computation were conducted using our IBSI-compliant in-house framework based on Python 3.6^35^.

### Robustness analysis

Feature robustness was assessed using the intraclass correlation coefficient (1, 1) (ICC)^16^, based on the assumption that test-retest images, as well as perturbations, possess no consistent bias. The highest possible ICC value is 1.00, which indicates that feature values are fully repeatable between test-retest images or perturbations. Lower values denote an increasing measurement variance with respect to the intra-patient variance, and thus lower repeatability.

The test-retest ICC was determined between both CT images, see Fig. 2. Perturbation ICCs were first computed separately for the test and retest images. Subsequently, perturbation ICCs were averaged over test and retest images to facilitate comparison with the test-retest ICC, as no consistent bias toward higher ICC values for one image set could be established (see Supplementary Note 6). The boundary values of the 95% confidence interval for perturbations were likewise averaged between test and retest images.

The 95% confidence interval of the ICC was used to determine robustness by comparison with a threshold of 0.90^17^. Thus, a feature is robust if CI ≥ 0.90, non-robust if CI < 0.90, and has an indeterminate robustness if the CI overlaps with the threshold.

Feature robustness was assessed using R 3.4.2^36^. ICCs and their confidence intervals were computed using code adapted from the psych R-package^37^.

## Supplementary information

Supplementary materials

Supplementary excel

## Data Availability

Source images for the NSCLC cohort are available from the Cancer Imaging Archive (10.7937/K9/TCIA.2015.U1X8A5NR). Due to complete anonymisation requirements under the General Data Protection Regulation of the European Union and data protection laws of the Federal Republic of Germany, source images for the HNSCC cohort can not be made publicly available. These data are available from the corresponding author on reasonable request, and pending approval by the local ethics committee. Only requests for academic use will be considered, as the patients did not consent to use of their data for non-academic, e.g. commercial, purposes. An anonymous ${\mathtt{csv}}$ table containing the intraclass correlation coefficients for all features and perturbation methods is made available with the article.

## References

[1] Kumar V (2012). Radiomics: the process and the challenges. Magn. Reson. Imaging.

[2] Lambin P (2012). Radiomics: Extracting more information from medical images using advanced feature analysis. Eur. J. Cancer.

[3] Mackin D (2015). Measuring Computed Tomography Scanner Variability of Radiomics Features. Investig. radiology.

[4] Yip SSF, Aerts HJWL (2016). Applications and limitations of radiomics. Phys. medicine biology.

[5] Lambin P (2017). Radiomics: the bridge between medical imaging and personalized medicine. Nat. reviews. Clin. oncology.

[6] Tixier F (2012). Reproducibility of tumor uptake heterogeneity characterization through textural feature analysis in 18F-FDG PET. J. nuclear medicine.

[7] Leijenaar RTH (2013). Stability of FDG-PET Radiomics features: An integrated analysis of test-retest and inter-observer variability. Acta Oncol..

[8] Balagurunathan Y (2014). Reproducibility and Prognosis of Quantitative Features Extracted from CT Images. Transl. oncology.

[9] van Velden FHP (2016). Repeatability of Radiomic Features in Non-Small-Cell Lung Cancer [(18)F]FDG-PET/CT Studies: Impact of Reconstruction and Delineation. Mol. imaging biology.

[10] Desseroit M-C (2017). Reliability of PET/CT Shape and Heterogeneity Features in Functional and Morphologic Components of Non-Small Cell Lung Cancer Tumors: A Repeatability Analysis in a Prospective Multicenter Cohort. J. nuclear medicine.

[11] van Timmeren JE (2016). Test-retest data for radiomics feature stability analysis: generalizable or study specific?. Tomogr..

[12] Hatt M (2015). 18F-FDG PET uptake characterization through texture analysis: investigating the complementary nature of heterogeneity and functional tumor volume in a multi-cancer site patient cohort. J. nuclear medicine.

[13] Shafiq-Ul-Hassan M (2017). Intrinsic dependencies of CT radiomic features on voxel size and number of gray levels. Med. physics.

[14] Mackin D (2017). Harmonizing the pixel size in retrospective computed tomography radiomics studies. PLOS ONE.

[15] Arel I, Rose DC, Karnowski TP (2010). Deep Machine Learning - A New Frontier in Artificial Intelligence Research. IEEE Comput. Intell. Mag..

[16] Shrout PE, Fleiss JL (1979). Intraclass correlations: Uses in assessing rater reliability. Psychol. Bull..

[17] Koo TK, Li MY (2016). A Guideline of Selecting and Reporting Intraclass Correlation Coefficients for Reliability Research. J. chiropractic medicine.

[18] Larue, R. T. H. M. *et al*. Influence of gray level discretization on radiomic feature stability for different CT scanners, tube currents and slice thicknesses: a comprehensive phantom study. *Acta oncologica* 1–10, 10.1080/0284186X.2017.1351624 (2017). 10.1080/0284186X.2017.1351624 28885084

[19] Mackin D (2018). Effect of tube current on computed tomography radiomic features. Sci. Reports.

[20] Midya A, Chakraborty J, Gönen M, Do RKG, Simpson AL (2018). Influence of CT acquisition and reconstruction parameters on radiomic feature reproducibility. J. Med. Imaging.

[21] Zhao B (2016). Reproducibility of radiomics for deciphering tumor phenotype with imaging. Sci. Reports.

[22] He L (2016). Effects of contrast-enhancement, reconstruction slice thickness and convolution kernel on the diagnostic performance of radiomics signature in solitary pulmonary nodule. Sci. reports.

[23] Pavic, M. *et al*. Influence of inter-observer delineation variability on radiomics stability in different tumor sites. Acta Oncol. 1–5, 10.1080/0284186X.2018.1445283 (2018). 10.1080/0284186X.2018.1445283 29513054

[24] Traverso A, Wee L, Dekker A, Gillies R (2018). Repeatability and Reproducibility of Radiomic Features: A Systematic Review. Int. journal radiation oncology, biology, physics.

[25] Zhao B (2009). Evaluating variability in tumor measurements from same-day repeat CT scans of patients with non-small cell lung cancer. Radiol..

[26] Zhao, B., Schwartz, L. H. & Kris, M. G. Data From RIDER Lung CT, 10.7937/K9/TCIA.2015.U1X8A5NR (2015).

[27] Löck S (2017). Residual tumour hypoxia in head-and-neck cancer patients undergoing primary radiochemotherapy, final results of a prospective trial on repeat FMISO-PET imaging. Radiother. oncology.

[28] Clark K (2013). The Cancer Imaging Archive (TCIA): maintaining and operating a public information repository. J. digital imaging.

[29] Zwanenburg, A., Leger, S., Vallières, M. & Löck, S. Image biomarker standardisation initiative. *eprint arXiv:1612.07003* [*cs.CV*] (2016).

[30] Vallières M (2018). Responsible Radiomics Research for Faster Clinical Translation. J. Nucl. Medicine.

[31] Achanta R (2012). SLIC superpixels compared to state-of-the-art superpixel methods. IEEE transactions on pattern analysis machine intelligence.

[32] Collewet G, Strzelecki M, Mariette F (2004). Influence of MRI acquisition protocols and image intensity normalization methods on texture classification. Magn. resonance imaging.

[33] Vallières M, Freeman CR, Skamene SR, El Naqa I (2015). A radiomics model from joint FDG-PET and MRI texture features for the prediction of lung metastases in soft-tissue sarcomas of the extremities. Phys. medicine biology.

[34] Vallières M (2017). Radiomics strategies for risk assessment of tumour failure in head-and-neck cancer. Sci. reports.

[35] Leger S (2017). A comparative study of machine learning methods for time-to-event survival data for radiomics risk modelling. Sci. Reports.

[36] R Core Team. R: A Language and Environment for Statistical Computing. Tech. Rep., Vienna, Austria (2017).

[37] Revelle, W. *psych: Procedures for Psychological, Psychometric, and Personality Research*. Northwestern University, Evanston, Illinois R package version 1.7.8. (2017).

