# Supplementary material for: Assessing robustness of radiomic features by image perturbation
Source: Sci Rep. 2019 Jan 24;9:614. doi: 10.1038/s41598-018-36938-4 (PMC6345842; doi:10.1038/s41598-018-36938-4)
Supplement: Supplementary file 1 — Supplementary materials [file 41598_2018_36938_MOESM1_ESM.pdf]

Assessing robustness of radiomic features by image perturbation

supplementary materials

Alex Zwanenburg, Stefan Leger, Linda Agolli, Karoline Pilz,
Esther G.C. Troost, Christian Richter and Steffen Löck

Supplementary note 1: image acquisition parameters

Computed tomography (CT) images were acquired for both the non-small-cell lung carcinoma (NSCLC) and head and neck squamous cell carcinoma (HNSCC) cohorts. For the NSCLC cohort, a second CT image was acquired 15 minutes after the first acquisition. The patient was asked to leave the table between the scans and was repositioned before the second image acquisition. For the HNSCC cohort a second CT image was recorded to determine attenuation corrections for positron emission tomography (PET). This PET-CT scan was recorded within 4 days after the original diagnostic CT scan. Acquisition parameters and characteristics are shown in Table S1.

**parameter**	**NSCLC**		**HNSCC**	
	CT 1	CT 2	CT 1	CT 2
number	31	31	19	19
scanner	GE Healthcare Lightspeed 16<br>GE Healthcare VCT	GE Healthcare Lightspeed 16<br>GE Healthcare VCT	Siemens Biograph 16	Siemens Biograph 16
tube voltage (kVp)	120	120	120	120
exposure (mAs)	8 (4-10)	8 (4-13)	36 (18-62)	9 (9-10)
reconstruction kernel	Lung	Lung	B31f	B19f
voxel spacing (x-axis; mm)	0.67 (0.51-0.90)	0.67 (0.51-0.91)	0.98	1.37
voxel spacing (y-axis; mm)	0.67 (0.51-0.90)	0.67 (0.51-0.91)	0.98	1.37
voxel spacing (z-axis; mm)	1.25	1.25	3.00 (2.00-3.00)	2.00
image noise (σ; HU)	29.3 (16.6-76.1)	28.4 (16.9-70.3)	4.1 (3.9-5.4)	4.2 (3.7-6.8)

Table S1 | Image acquisition parameters and characteristics for both NSCLC and HNSCC image data sets. Parameters were determined from the CT slices that contain portions of the gross tumour volume (GTV) region of interest. Numeric parameters are presented as *median* (*range*), unless only one value was found within the cohort. Image noise was calculated using Chang's method[1] and represented by its standard deviation σ (supplementary note 4). *kVp*: peak kilovoltage; *HU*: Hounsfield unit

Supplementary note 2: pre-interpolation low-pass filtering

Image features are computed from voxels with uniform dimensions. In this work, features are computed with voxel spacings of 1, 2, 3 and 4 mm. The in-plane original spacing of the CT images is between 0.51 and 1.37 mm. We therefore need to down-sample images, which may cause image artefacts through aliasing and thus reduce feature robustness. In signal analysis, a signal may contain only frequencies up to half the sample frequency (the Nyquist frequency ω_N) of the down-sampled signal to avoid artefacts. Signals are therefore low-pass filtered before down-sampling to suppress high frequency contents. The same concept applies to images as well. However, application of low-pass filters in radiomics is often neglected, despite the beneficial effect on feature robustness[2].

We use a low-pass Gaussian filter before interpolation scipy.ndimage.gaussian_filter. The Gaussian function $g(x)$ is defined as:

$$g(x) = \frac{1}{\sigma\sqrt{2\pi}} \mathrm{e}^{-\frac{x^2}{2\sigma^2}},$$

with σ the standard deviation, or width, of the distribution. σ is an input parameter for the Gaussian filter for which optimal settings have not been established. σ moreover needs to be defined with respect to the typically non-uniformly spaced coordinate grid system of the original image and is thus specified separately for each axis.

Fourier theory allows us to set σ based on the Nyquist frequency. The Fourier transform of the Gaussian function g is[3]:

$$G(\omega) = \mathrm{e}^{-\frac{\omega^2\sigma^2}{2}},$$

with ω being a frequency. An ideal low-pass filter will maintain all frequencies $\omega < \omega_N$, and remove frequencies $\omega \geq \omega_N$ completely. However, ideal filters do not exist and a compromise is required between the desired attenuation of high-frequency content and the unwanted attenuation of low-frequency content. We define a smoothing parameter β, with $0 < \beta \leq 1$, for the Fourier transformed Gaussian at $\omega = \omega_N$:

$$G(\omega_N) = \mathrm{e}^{-\frac{\omega_N^2\sigma^2}{2}} = \beta \tag{1}$$

The Nyquist frequency ω_N may be expressed in terms of voxel spacing. For instance, we have a one-dimensional array of voxels with spacing d_1. We want to sample this array to spacing d_2. The sampling frequency is then $\omega_s = d_1/d_2$, which leads to the Nyquist frequency $\omega_N = \omega_s/2 = d_1/(2d_2)$.

We now solve equation (1) for σ:

$$\begin{aligned}
&\mathrm{e}^{-\frac{\omega_N{}^2\sigma^2}{2}} = \beta \Leftrightarrow \\
&\ln(\beta) = -\frac{\omega_N{}^2\sigma^2}{2} \Leftrightarrow \\
&\sigma^2 = -\frac{2\ln(\beta)}{\omega_N{}^2} = -8\left(\frac{d_2}{d_1}\right)^2 \ln(\beta) \Leftrightarrow \\
&\sigma = -2\frac{d_2}{d_1}\sqrt{2\ln(\beta)}
\end{aligned}$$

We assess different parameter settings for β, namely $\beta = \{0.50, 0.70, 0.80, 0.85, 0.90,$

$0.93, 0.95, 0.97\}$, as well as no low-pass filtering. Test-retest intraclass correlation coefficients (ICC (1,1)) and their 95% confidence intervals (CI) are calculated on both test-retest cohorts[4]. The ICCs are used to determine the number of robust features and to show the ICC distribution. In addition, the distribution of the width of the ICC 95% confidence intervals is assessed.

Example images of an interpolated slice acquired from an NSCLC and an HNSCC patient are shown in Figures S1 and S2, respectively. Down-sampling without interpolation caused visible image artefacts. On the other hand, images that are smoothed with a wide Gaussian low-pass filter (low β value) lack detail.

The percentage of robust features according to the test-retest ICC is shown in Figure S3. For the NSCLC cohort, even very light smoothing ($\beta = 0.97$) increases the percentage of robust features from 59.0% to 75.9%. With lower β-values, this percentage does not change, nor does the distribution of ICCs (Figure S4) or the distribution of ICC CI widths (Figure S5). For very low β-values, the ICC distribution for NSCLC may be less stable.

For the HNSCC cohort, the percentage of robust features increases with decreasing β, which is also reflected in the ICC distribution. In particular, even very mild smoothing ($\beta = 0.97$) increased the median ICC from 0.63 to 0.76. When only features computed with minimal down-sampling are considered (1 mm), $\beta = 0.97$ reduced the median ICC from 0.72 to 0.65, and only recovered at $\beta = 0.93$. The same may be observed for the ICC CI width, which was increased for $\beta = 0.97$. A smoothing parameter value between $\beta = 0.93$ (robust features: 34.0%; median ICC: 0.85; median CI width: 0.29) and $\beta = 0.90$ (robust features: 43.0%; median ICC: 0.88; median CI width: 0.23) offers a good compromise between aliasing and lack of image details.

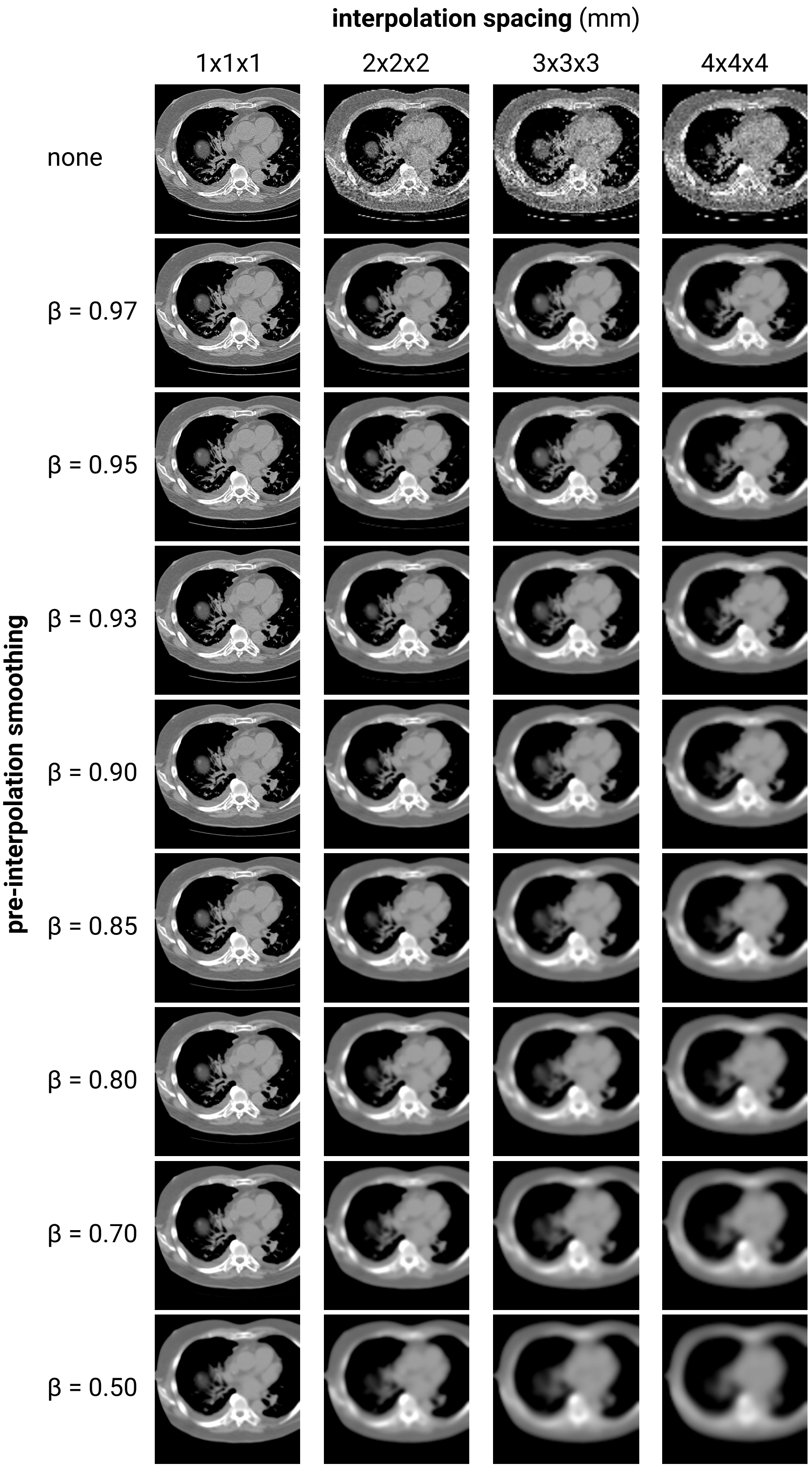

Figure S1 | Effect of smoothing and interpolation on a CT slice of an NSCLC patient. A Gaussian smoothing filter for the given β-values was applied before interpolation. Afterwards, tri-linear interpolation was conducted to resample to uniform voxel spacing (in mm). All slices are shown at the same size for comparison, and intensities were windowed between $[-400, 300]$ HU.

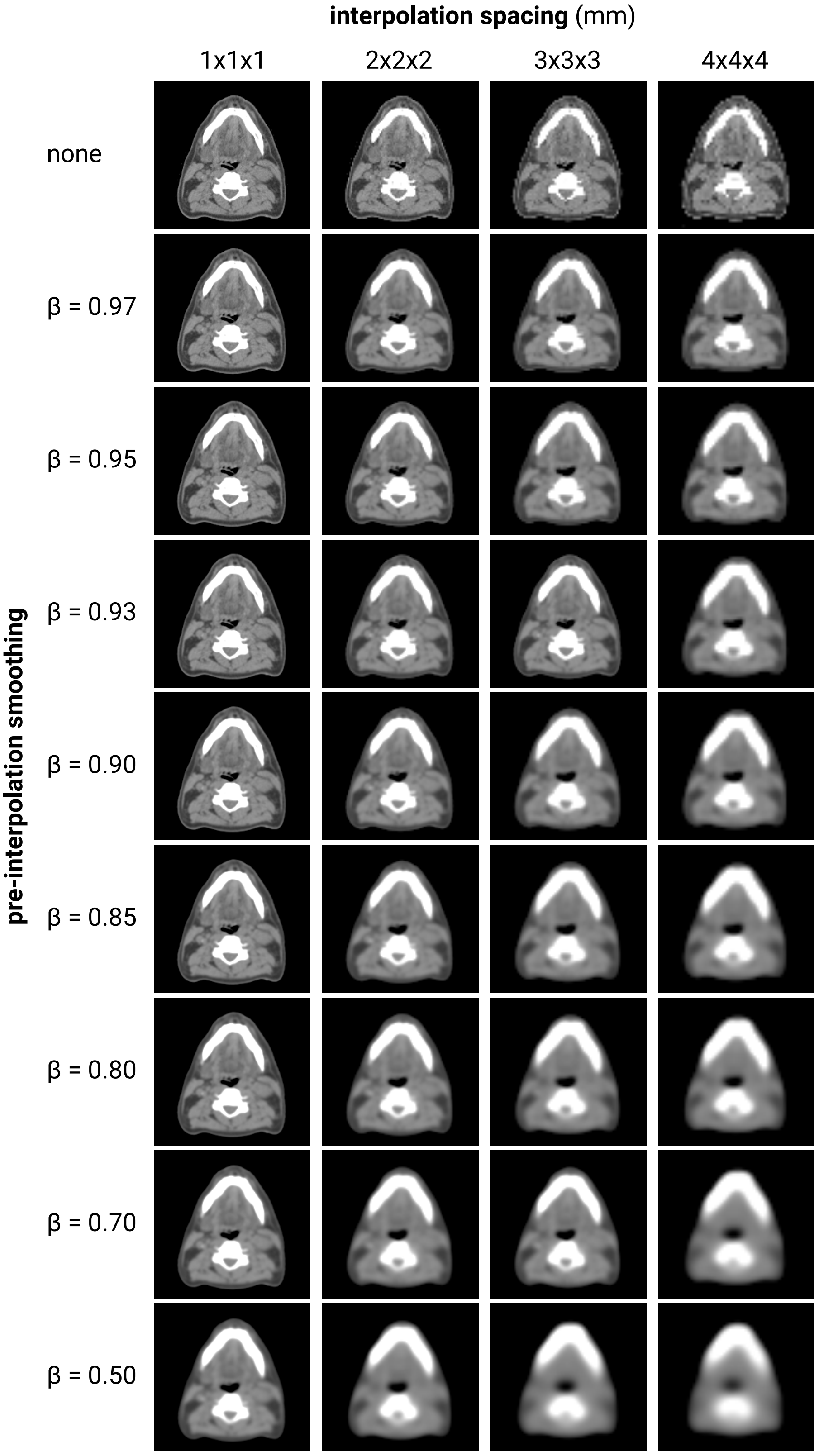

Figure S2 | Effect of smoothing and interpolation on a CT slice of an HNSCC patient. A Gaussian smoothing filter was applied before interpolation for the given β-values. Afterwards, tri-linear interpolation was conducted to resample to uniform voxel spacing (in mm). All slices are shown at the same size for comparison, and intensities were windowed between $[-220, 250]$ HU.

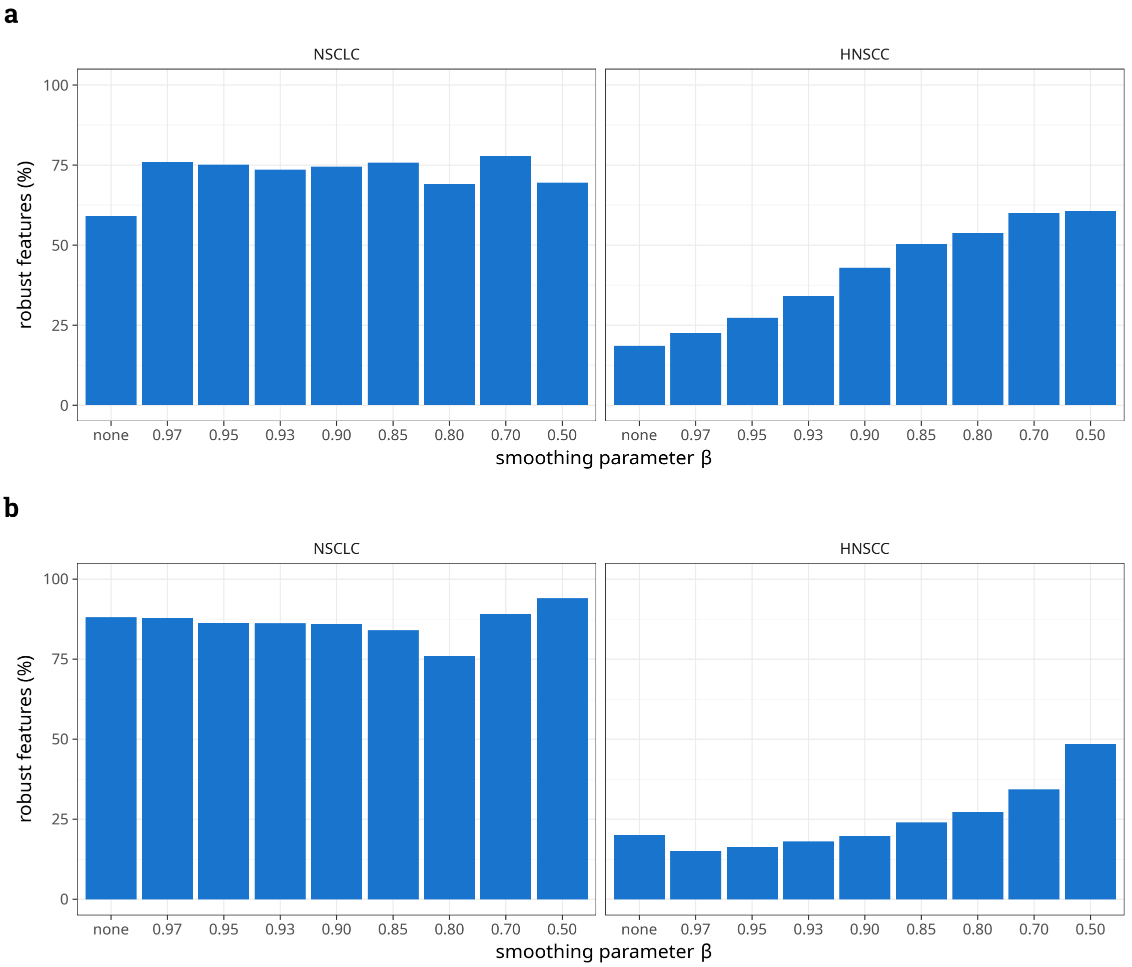

Figure S3 | Fraction of robust features according to the test-retest intraclass correlation coefficient (ICC (1,1)) for a pre-interpolation Gaussian smoothing parameter β. A feature was considered robust if ICC $\geq$ 0.90. Lower β-values indicate stronger smoothing. The fraction of robust features is shown for all features (**a**) and for features acquired using a uniform spacing of 1 mm (**b**).

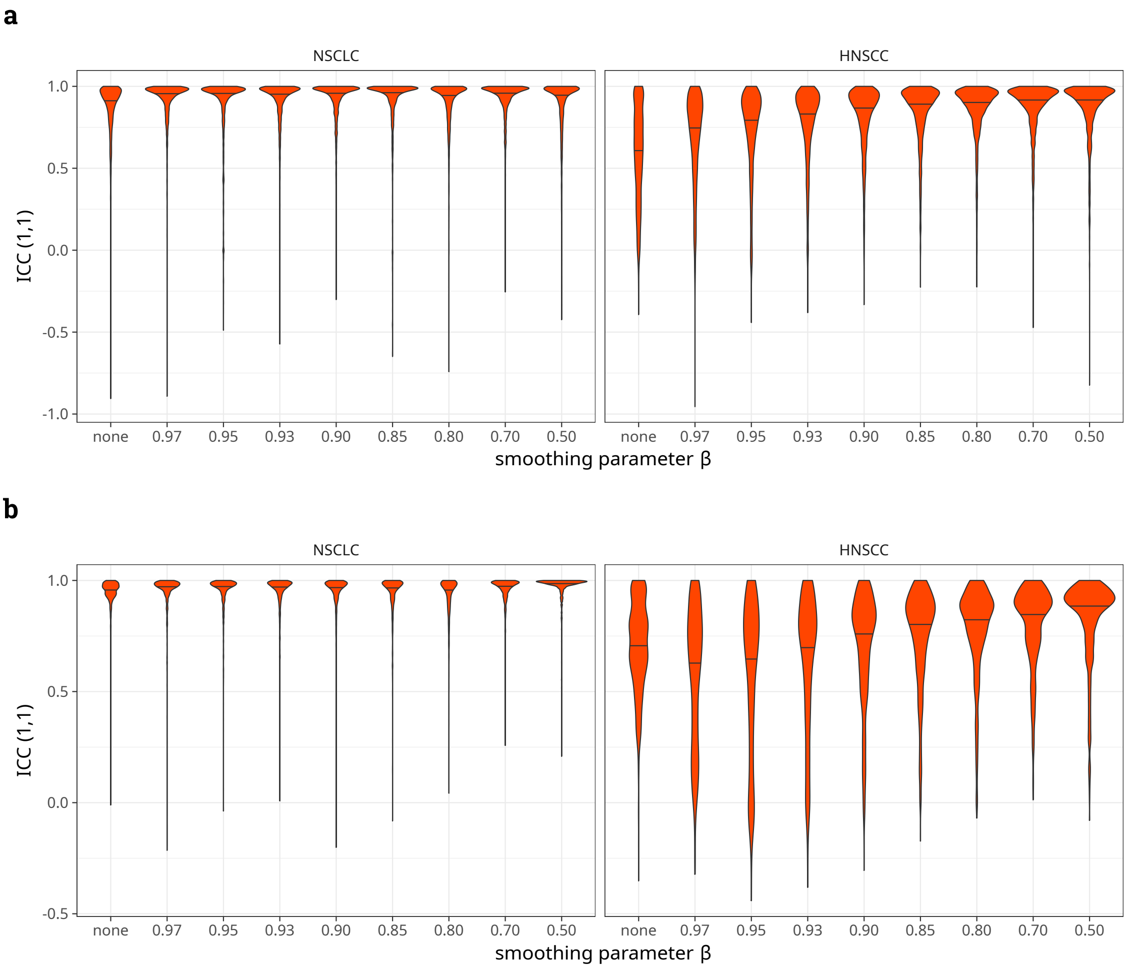

Figure S4 | Distribution of test-retest intraclass correlation coefficients (ICC (1,1)) for a pre-interpolation Gaussian smoothing parameter β. Lower β-values indicate stronger smoothing. The areas of the distributions were normalised. The median ICC in each distribution is indicated by a horizontal line. ICC distributions are shown for all features (**a**) and for features acquired using a uniform spacing of 1 mm (**b**).

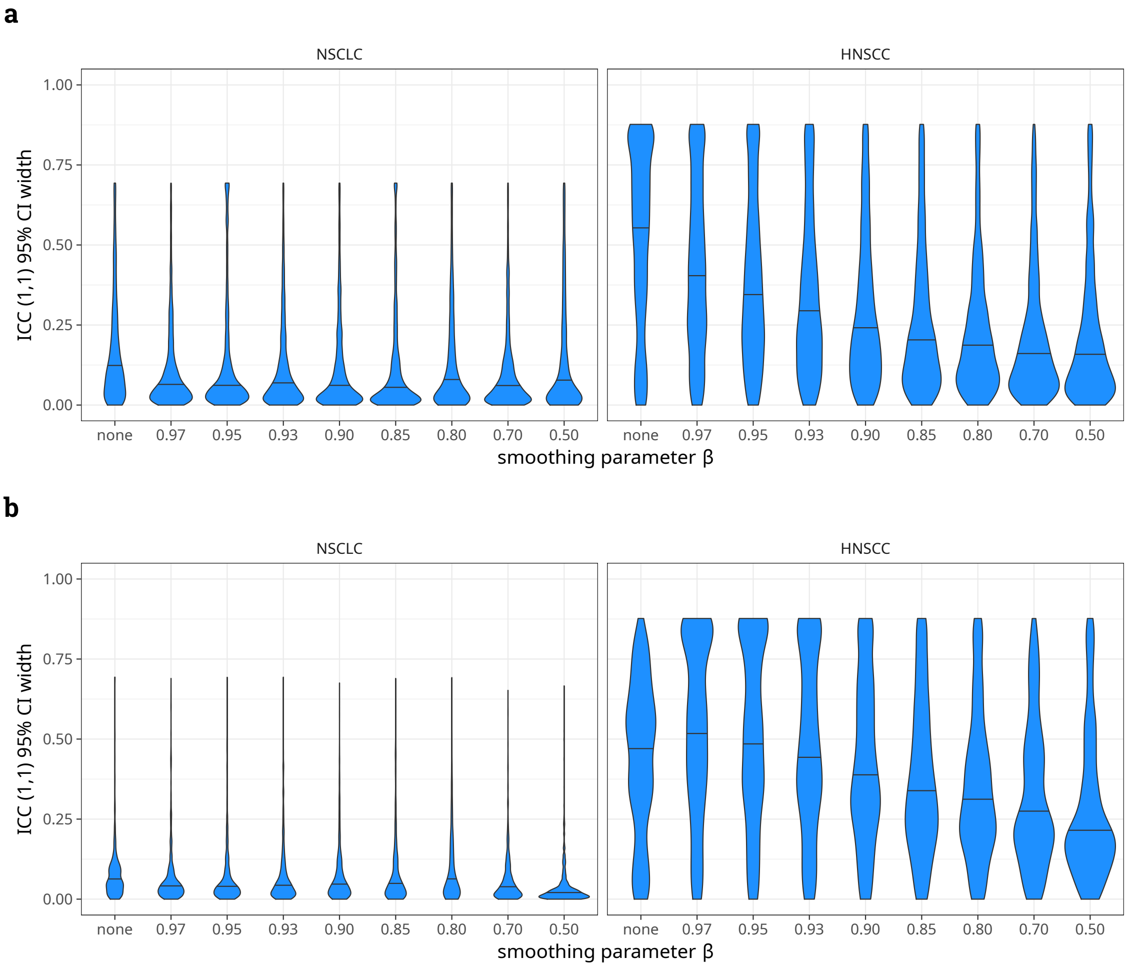

Figure S5 | Distribution of the 95 % confidence interval (CI) widths of the test-retest intraclass correlation coefficients (ICC (1,1)) for a pre-interpolation Gaussian smoothing parameter β. Higher CI widths indicate larger variance in feature values between test and retest images. Lower β-values indicate stronger smoothing. The areas of the distributions were normalised. The median 95% CI width is indicated in each distribution by a horizontal line. ICC CI width distributions are shown for all features (**a**) and for features acquired using a uniform spacing of 1 mm (**b**).

Supplementary note 3: image features

All image features were extracted according to the definitions provided by the Image Biomarker Standardisation Initiative[5]. Intensity-volume histogram-based features were calculated for the 10th, 25th, 50th, 75th and 90th intensity and volume fraction percentiles. Moran's I index and Geary's C measure were approximated by repeatedly selecting 100 voxels from the ROI at random and computing these metrics until the standard error of the mean decreased below 0.002. A total of 4032 features were computed, see Table S2.

The following specific parameters were used to compute image features:

- **Morphology**: the surface mesh was constructed using the *Marching Cubes* algorithm, with an iso-level of 0.5[6,7].
- **Intensity-volume histogram**: the intensity volume histogram was constructed as for images with discrete, defined (non-arbitrary) image values[5].
- **Grey level co-occurrence matrix**: grey level co-occurrence matrices (GLCM) were calculated in 3D for 13 directions, with Chebyshev distance $\delta = 1$. GLCM were symmetric and not distance-weighted. GLCM features were first calculated for every GLCM, and subsequently averaged.
- **Grey level run length matrix**: grey level run length matrices (GLRLM) were calculated in 3D for 13 directions. GLRLM were not distance-weighted. GLRLM features were first calculated for every GLRLM, and subsequently averaged.
- **Grey level size zone matrix**: a single grey level size zone matrix was calculated for the entire 3D volume.
- **Grey level distance zone matrix**: a single grey level distance zone matrix was calculated for the entire 3D volume.
- **Neighbourhood grey tone difference matrix**: a single neighbourhood grey tone difference matrix was calculated for the entire 3D volume, with Chebyshev distance $\delta = 1$.
- **Neighbouring grey level dependence matrix**: a single neighbouring grey level dependence matrix was calculated for the entire 3D volume, with Chebyshev distance $\delta = 1$ and coarseness parameter $\alpha = 0$.

Intensity histogram, grey level co-occurrence matrix, grey level run length matrix, grey level size zone matrix, grey level distance zone matrix, neighbourhood grey tone difference matrix and neighbouring grey level dependence matrix-based features required image discretisation prior to computation, which was conducted using two methods, with four settings each: a fixed bin number method with 8, 16, 32 or 64 bins; or a fixed bin width method with bins that were 6, 12, 18 or 24 Hounsfield units (HU) wide. For the fixed bin number method, the edge of the first bin coincided with the lowest intensity of the voxels included in the intensity mask. For the fixed bin width method, the lower edge of the first bin coincided with the lower edge of the re-segmentation range applied during image processing (NSCLC: -300 HU; HNSCC: -150 HU).

feature family	base #	multipl.	total #
morphology	29		29
local intensity	2		2
intensity-based statistics	18		18
intensity histogram	23	×8	184
intensity-volume histogram	15		15
grey level co-occurrence matrix	25	×8	200
grey level run length matrix	16	×8	128
grey level size zone matrix	16	×8	128
grey level distance zone matrix	16	×8	128
neighbourhood grey tone difference matrix	5	×8	40
neighbouring grey level dependence matrix	17	×8	136
total			1008

Table S2 | Feature families and the number of computed features. Many features require discretisation prior to computation. As two discretisation methods with four bin size settings each were evaluated, the total number of such features is 8 times the number of base definitions. The grand total of features is 4032, due to computation for four different interpolation spacings.

Supplementary note 4: Image perturbation algorithms

This note provides additional information with regards to the implementation of the image perturbation algorithms. The algorithms were implemented in Python 3.6.1 (Python Software Foundation, Beaverton, Oregon, USA, https://www.python.org/). The implementation drew on functionality offered by the following libraries:

- NumPy 1.13.3[8,9], referred to as numpy.
- SciPy 0.19.1[8,9], referred to as scipy.
- scikit-image 0.13.1[10], referred to as skimage.
- PyWavelets 0.5.2[11], referred to as pywt.

Specific functions from the libraries mentioned above are referred to in text.

Rotation

Image rotations emulate changes due to different patient positioning. Image features should be robust against such perturbations to be reproducible.

The image is rotated in-plane around the z-axis by an angle θ. Rotation was performed using the scipy.ndimage.rotate function, which implements rotation as an affine transformation. Bi-linear sampling is used to determine intensities in the rotated image. After rotation, intensities are rounded to the nearest integer value to conform with the expected integer Hounsfield units in CT.

The ROI mask is rotated in the same way as the image. However, the threshold for partial volume fractions in the mask is only applied after the interpolation step in the image processing scheme.

Noise

Noise affects voxel intensities. Reproducible features should be robust to the noise present in an image. Perturbation by noise addition therefore follows two steps. First, the noise-dependent intensity variance is determined. Secondly, noise drawn from a Gaussian distribution with the same variance is added to the image.

The method of Chang *et al.*[1,12] is used to determine noise variance. In short, the image $\mathbf{I}$ is filtered in both the x and y direction in the image plane (z being the axis along which the image slices are stacked) using a one-dimensional stationary *coiflet-1* wavelet high-pass filter, pywt.Wavelet("coif1").dec_hi. The filter convolution was implemented using the scipy.ndimage.convolve1d function. This cascade filter operation yields $\mathbf{I}_{\text{diff}}$. Subsequently, the noise level is estimated as:

$$\sigma_{\text{noise}} = \frac{\text{median}\left(|\mathbf{I}_{\text{diff}}|\right)}{0.6754}.$$

Subsequently, for every image voxel random noise from a normal (Gaussian) distribution with mean 0 and standard deviation $\sigma = \sigma_{\text{noise}}$ is generated (numpy.random.normal), and added. After noise addition, intensities are rounded to the nearest integer value to conform with the expected integer Hounsfield units in CT.

Noise variance is determined on the original image data, before any rotation, translation or other operation occurs. In the image processing scheme, noise addition takes place after rotation of the image, if applicable.

Translation

Translation, like rotation, emulates changes due to different patient positioning. Translation was performed concurrently with interpolation, i.e. the interpolation grid was shifted off-centre by the provided translation fraction η multiplied by the interpolation grid spacing. Translation was conducted along the x, y and z axes. Translation and interpolation was conducted with tri-linear approximation using the scipy.ndimage.map_coordinates function.

Volume adaptation

Shrinking or growing the segmentation mask is a method to mimic variance in expert delineations. For example, Fotina *et al.* reported a mean coefficient of variance in volume of 14.9% (range:$[4.4, 29.3]$%) in CT-based expert delineations for lung and prostate cancer. The proposed method for volume adaptation is simple and intensity-agnostic, and is conducted as follows:

1. Approximate the volume V_0 of the ROI $\mathbf{R}_0$ by counting the number of voxels in the mask.
2. Calculate the volume of the ROI after adaptation (rounded down towards the nearest integer) by $V_a = \lfloor V_0(1+\tau) \rfloor$, with τ the required growth/shrinkage fraction. $\tau > 0.0$ indicates volume growth, and $\tau < 0.0$ indicates shrinkage.
3. Define a geometric structure element that includes all voxels within Manhattan distance 1 (i.e. a centre voxel and its directly adjacent neighbours). We used the scipy.ndimage.generate_binary_structure(3,1) function.

4. Initialise a place-holder for an adapted mask $\mathbf{R}_p$ with volume V_p by copying the original ROI and its volume. This place-holder is used to track the volume and mask over iterative adaptations.

5. Iterate the mask shrinkage/growth process until the loop breaks:

   (a) If $\tau > 0.0$ dilate the mask (scipy.ndimage.binary_dilation) once, using the structure element defined in step 3.

   (b) If $\tau < 0.0$ erode the mask (scipy.ndimage.binary_erosion) once, using the structure element defined in step 3.

   (c) Approximate the volume V_n of the newly adapted mask $\mathbf{R}_n$ by counting the number of voxels in the mask.

   (d) If $V_n = 0.0$ break from the loop.

   (e) If $\tau > 0.0$ and $V_n > V_a$ break from the loop.

   (f) If $\tau < 0.0$ and $V_n < V_a$ break from the loop.

   (g) Replace the previous place-holder mask by setting $\mathbf{R}_p = \mathbf{R}_n$. This is done until the final growth/shrinkage iteration, when one of the conditions in steps d-f was satisfied.

6. If $V_n \neq V_a$, $\mathbf{R}_n$ contains either too many ($\tau > 0.0$) or too few ($\tau < 0.0$) voxels. A limited number of voxels should be added to or removed from the mask $\mathbf{R}_p$ to complete the adaptation. Practically, we update the rim formed by the disjunctive union of $\mathbf{R}_p$ and $\mathbf{R}_n$, i.e. $\mathbf{R}_r = \mathbf{R}_n \ominus \mathbf{R}_p$:

   (a) Determine the number of voxels to be added/removed from the mask: $N = |V_a - V_p|$.

   (b) Find rim $\mathbf{R}_r$ by logical XOR comparison of $\mathbf{R}_n$ and $\mathbf{R}_p$ (numpy.logical_xor).

   (c) Select N voxels from the rim at random, without replacement (numpy.random.choice).

   (d) If $N > 0$ and $\tau > 0.0$ add the N voxels to mask $\mathbf{R}_p$.

   (e) If $N > 0$ and $\tau < 0.0$ remove the N voxels from mask $\mathbf{R}_p$.

7. Volume adaptation ends. The mask $\mathbf{R}_p$ defines the perturbed region of interest.

Contour randomisation

Multiple image segmentations are required for randomising the contour of the region of interest. Creating multiple segmentations usually requires delineation by multiple experts. However, for larger quantities of image data, the creation of multiple manual delineations is extremely time-consuming and unfeasible in practice. An automated contour randomisation is therefore required. We use supervoxel-based segmentation algorithm for randomising contours. Supervoxels are connected clusters of voxels with similar intensity characteristics. To create a random contour, we compare supervoxels with a single segmentation delineated by an expert. The region of interest (ROI) is then randomised based on the overlap of supervoxels with the expert contour. Multiple algorithms produce supervoxels. We used the *simple linear iterative clustering* (SLIC) algorithm as it efficiently produces compact, contiguous supervoxels[13]. This algorithm was provided through the skimage.segmentation.slic_superpixels function.

Contour randomisation is conducted as follows:

1. Both the image and the region of interest (ROI) mask are cropped to 25 mm around the ROI bounding box to limit computational costs.
2. The intensities of the cropped image stack $\mathbf{I}$ are translated to a $[0,1]$ range:
   (a) Intensities $I_j \in \mathbf{I}$ are first restricted to range $\mathbf{r}$, which is based on the range used for ROI re-segmentation (Table 2 in main manuscript). The intensity range extends the re-segmentation range by 10% at both the upper (g_u) and lower (g_l) boundaries:

   $$\mathbf{r} = [g_l - 0.1 \cdot (g_u - g_l), g_u + 0.1 \cdot (g_u - g_l)] = [r_1, r_2]$$

   All intensity values outside range $\mathbf{r}$ are replaced by the nearest valid intensity:

   $$I_j = \begin{cases} r_1, & I_j < r_1 \\ r_2, & I_j > r_2 \\ I_j, & \text{otherwise} \end{cases}$$

   (b) Intensities are then mapped to the $[0,1]$ range by a simple transformation:

   $$I_{j,s} = \frac{I_j - r_1}{r_2 - r_1}$$

3. The number of supervoxels is estimated so that on average each supervoxel occupies 0.5 cm^3:

   $$N_{\text{sx,est}} = \left\lceil \frac{N_v V_{vox}}{0.5} \right\rceil,$$

   with N_v the number of voxels in $\mathbf{I}_s$ and V_{vox} the volume of each voxel (in cm^3). $\lceil \ldots \rceil$ denotes a ceiling operation that rounds the fraction up towards the nearest integer.
4. The SLIC algorithm pre-processes $\mathbf{I}_s$ by applying a Gaussian smoothing filter. The filter scaling parameter σ is set to the uniform voxel spacing (1,2,3 or 4 mm).
5. SLIC is performed, using the skimage.segmentation.slic_superpixels function, with filter scaling parameter σ, the estimated number of supervoxels $N_{\text{sx,est}}$, compactness $\beta = 0.05$, and by allowing supervoxels to vary in size between 0.25 cm^3 and 1.5 cm^3. This results in a mask $\mathbf{S}$ that labels supervoxels in $\mathbf{I}_s$. A total N_{sx} supervoxels are labelled.
6. The overlap η_k of the different supervoxels $\mathbf{S}_k \subset \mathbf{S}$, where $k = 1, \ldots, N_{\text{sx}}$, and the morphological ROI mask $\mathbf{R}$ defined by the expert is determined as follows:
   (a) The number of voxels m_k labelled by supervoxel $\mathbf{S}_k$ is counted.
   (b) The number of voxels $m_{\eta,k}$ in the intersection of the ROI mask and the supervoxel, $\mathbf{R} \cap \mathbf{S}_k$ is counted.
   (c) The overlap fraction for supervoxel k is then defined as:

   $$\eta_k = m_k / m_{\eta,k}$$

   By definition, $0.0 \leq \eta_k \leq 1.0$.
7. Subsequently, supervoxels are selected to form a new supervoxel-based ROI mask $\mathbf{R}_{\text{sx}}$, as follows:

(a) To ensure that the new ROI mask will not remain empty, i.e. $\mathbf{R}_{\text{sx}} \neq \emptyset$, the supervoxel with the highest overlap is always selected, regardless of the actual overlap.

(b) Additionally, all supervoxels with overlap $\eta \geq 0.90$ are always selected.

(c) All supervoxels with overlap $\eta < 0.20$ are never selected.

(d) All supervoxels with overlap $0.20 \leq \eta \leq 0.90$ are randomly selected. For each supervoxel k, a random number is drawn uniformly from the interval $[0, 1]$, i.e. $x_k \sim \mathcal{U}(0, 1)$, using the numpy.random.random function. If $x_k \leq \eta_k$, the supervoxel is added to the mask. Thus, selection probabilities for such supervoxels are equal to the overlap.

(e) The resulting supervoxel-based ROI mask $\mathbf{R}_{\text{sx}}$ is morphologically closed using the scipy.ndimage.binary_closing function with a geometric structure element that includes all voxels within Manhattan distance 1 (i.e. a centre voxel and its directly adjacent neighbours).

8. Contour randomisation ends. The mask $\mathbf{R}_{\text{sx}}$ defines the perturbed region of interest.

Supplementary note 5: image perturbation settings

Eighteen perturbation chains were constructed from the five basic perturbations. Rotation was performed by rotating the image around the z-axis by an angle θ. Translation was performed by shifting the voxel grid by a fraction η of the voxel spacing. If more than one fraction was provided, translations were performed using all permutations of η and the three primary axes. Thus, we performed eight permutations if two fractions η were provided, and 27 for three fractions. Volume adaptation required a shrinkage/growth fraction τ, with negative values indicating shrinkage and positive values growth of the mask. Noise adaptation and contour randomisation did not require additional settings, but could be repeated.

We perturbed images using every permutation of the settings of a perturbation chain. Several combinations of perturbations were not tested as the number of permutations was excessive. In particular, chains that combined rotation, translation and volume adaptation were not tested, as a typical set of 5 rotation angles, 2 translation fractions and 5 volume growth/shrinkage factors would lead to 200 permutations. We defined perturbation chains that lead to roughly 30 permutations to limit the effect of sample size on the intraclass correlation coefficient. In addition, we did not test every possible perturbation chain that included noise addition, as noise addition had a marginal effect if used in combination with other perturbations.

The following perturbations were defined, with m the total number of perturbed images generated:

1. Rotation (R, $m = 27$)
   - *rotation*: $\theta = \{-13^\circ, -12^\circ, \dots, 13^\circ\}$
2. Noise addition (N, $m = 30$)
   - *noise addition*: 30 repetitions
3. Translation (T, $m = 27$)
   - *translation*: $\eta = \{0.0, 0.333, 0.667\}$
4. Volume adaptation (V, $m = 29$)
   - *volume adaptation*: $\tau = \{-0.28, -0.26, \dots, 0.28\}$
5. Contour randomisation (C, $m = 30$)
   - *contour randomisation*: 30 repetitions
6. Rotation and translation (RT, $m = 32$)
   - *rotation*: $\theta = \{-6^\circ, -2^\circ, 2^\circ, 6^\circ\}$
   - *translation*: $\eta = \{0.25, 0.75\}$
7. Rotation, noise addition and translation (RNT, $m = 32$)
   - *rotation*: $\theta = \{-6^\circ, -2^\circ, 2^\circ, 6^\circ\}$
   - *noise addition*: 1 repetition
   - *translation*: $\eta = \{0.25, 0.75\}$
8. Rotation and volume adaptation (RV, $m = 30$)
   - *rotation*: $\theta = \{-10^\circ, -6^\circ, -2^\circ, 2^\circ, 6^\circ, 10^\circ\}$
   - *volume adaptation*: $\tau = \{-0.2, -0.1, 0.0, 0.1, 0.2\}$
9. Rotation and contour randomisation (RC, $m = 27$)
   - *rotation*: $\theta = \{-13^\circ, -12^\circ, \dots, 13^\circ\}$

- *contour randomisation*: 1 repetition

10. Translation and volume adaptation (TV, $m = 40$)
    - *translation*: $\eta = \{0.25, 0.75\}$
    - *volume adaptation*: $\tau = \{-0.2, -0.1, 0.0, 0.1, 0.2\}$
11. Translation and contour randomisation (TC, $m = 27$)
    - *translation*: $\eta = \{0.0, 0.333, 0.667\}$
    - *contour randomisation*: 1 repetition
12. Rotation, translation, and contour randomisation (RTC, $m = 32$)
    - *rotation*: $\theta = \{-6^\circ, -2^\circ, 2^\circ, 6^\circ\}$
    - *translation*: $\eta = \{0.25, 0.75\}$
    - *contour randomisation*: 1 repetition
13. Rotation, noise addition, translation, and contour randomisation (RNTC, $m = 32$)
    - *rotation*: $\theta = \{-6^\circ, -2^\circ, 2^\circ, 6^\circ\}$
    - *noise addition*: 1 repetition
    - *translation*: $\eta = \{0.25, 0.75\}$
    - *contour randomisation*: 1 repetition
14. Volume adaptation and contour randomisation (VC, $m = 30$)
    - *volume adaptation*: $\tau = \{-0.2, -0.1, 0.0, 0.1, 0.2\}$
    - *contour randomisation*: 6 repetitions
15. Rotation, volume adaptation and contour randomisation (RVC, $m = 30$)
    - *rotation*: $\theta = \{-10^\circ, -6^\circ, -2^\circ, 2^\circ, 6^\circ, 10^\circ\}$
    - *volume adaptation*: $\tau = \{-0.2, -0.1, 0.0, 0.1, 0.2\}$
    - *contour randomisation*: 1 repetition
16. Rotation, noise addition, volume adaptation and contour randomisation (RNVC, $m = 30$)
    - *rotation*: $\theta = \{-10^\circ, -6^\circ, -2^\circ, 2^\circ, 6^\circ, 10^\circ\}$
    - *noise addition*: 1 repetition
    - *volume adaptation*: $\tau = \{-0.2, -0.1, 0.0, 0.1, 0.2\}$
    - *contour randomisation*: 1 repetition
17. Translation, volume adaptation and contour randomisation (TVC, $m = 40$)
    - *translation*: $\eta = \{0.25, 0.75\}$
    - *volume adaptation*: $\tau = \{-0.2, -0.1, 0.0, 0.1, 0.2\}$
    - *contour randomisation*: 1 repetition
18. Noise addition, translation, volume adaptation and contour randomisation (NTVC, $m = 40$)
    - *noise addition*: 1 repetition
    - *translation*: $\eta = \{0.25, 0.75\}$
    - *volume adaptation*: $\tau = \{-0.2, -0.1, 0.0, 0.1, 0.2\}$
    - *contour randomisation*: 1 repetition

Supplementary note 6: Robustness differences between perturbed test and retest images

Perturbation ICCs are averaged between test and retest images for easier comparison with test-retest ICCs. To verify that there is no significant bias in perturbation ICC towards one image, we first calculated the difference between the perturbation ICCs of the same feature for every feature. Subsequently, we calculate the mean μ and standard deviation σ of the differences, and perform a one-sided location test against mean 0:

$$z = \sqrt{n}\frac{\mu - 0}{\sigma}$$

$|z| \geq 1.96$ corresponds to a significance level $p \leq 0.05$. The ICC difference of each feature can not be considered independent as many features are known to be correlated, which affects the choice for n. Hence, we chose $n = 1$ (complete pooling), instead of $n = 4032$ for independent samples (Z-test). None of perturbations were distributed significantly from 0. The distribution of perturbation ICC differences is shown in Figure S6.

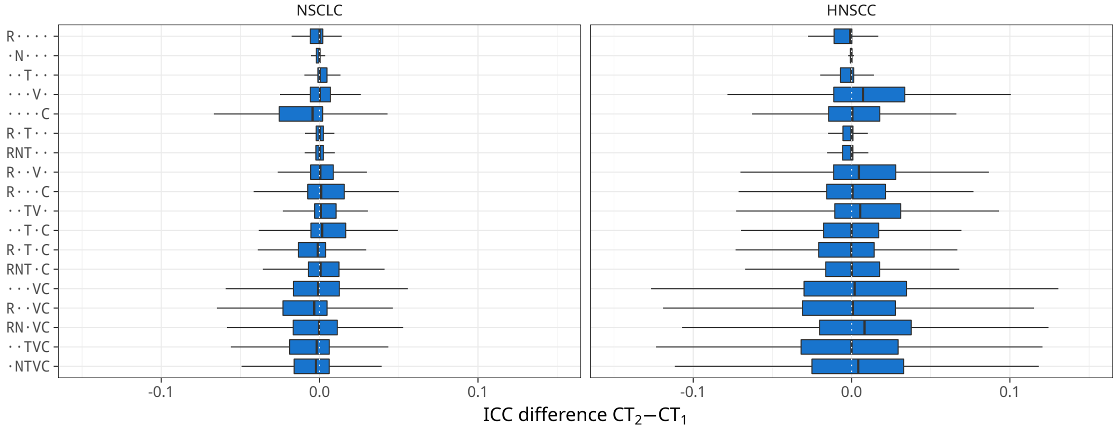

Figure S6 | Box plots of the differences in intraclass correlation coefficient (ICC) between test (CT_1) and retest (CT_2) data sets for the perturbation chains. The boxes cover the interquartile range (IQR), and the median ICC is indicated. The whiskers of each plot extend to 1.5 times the IQR.

Supplementary note 7: Robustness under different image parameters

NSCLC and HNSCC cohorts have a different overall test-retest robustness. The particular range of image parameters for interpolation and discretisation could be a principal cause for these differences. Test-retest robustness fractions as a function of interpolated voxel size are shown in Figure S7. Test-retest robustness fractions for features computed using fixed bin size and fixed bin number discretisation methods are shown in Figures S8 and S9, respectively.

There does not appear to be a specific parameter setting that is causing the difference in the number of robust features between both cohorts. Moreover, both cohorts have an increasing amount of features for which robustness could not be accurately determined with increasing voxel spacing, though the effect is more noticeable in the HNSCC cohort.

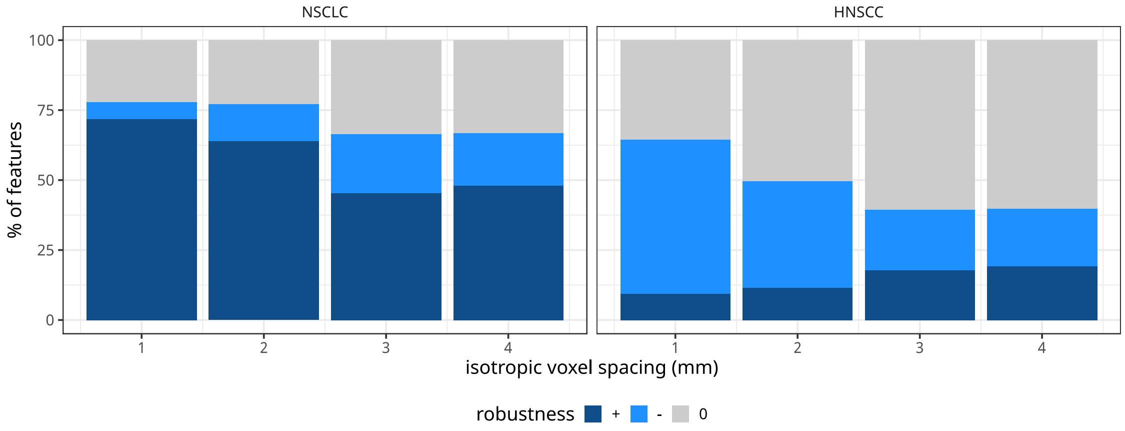

Figure S7 | Fraction of robust features in the test-retest set for different interpolated isotropic voxel spacings. Robustness was determined using the 95% confidence interval (CI) of the intraclass correlation coefficient. Features with CI ≥ 0.90 were considered to be robust (+), CI < 0.90 non-robust (-), and indeterminate (0) otherwise.

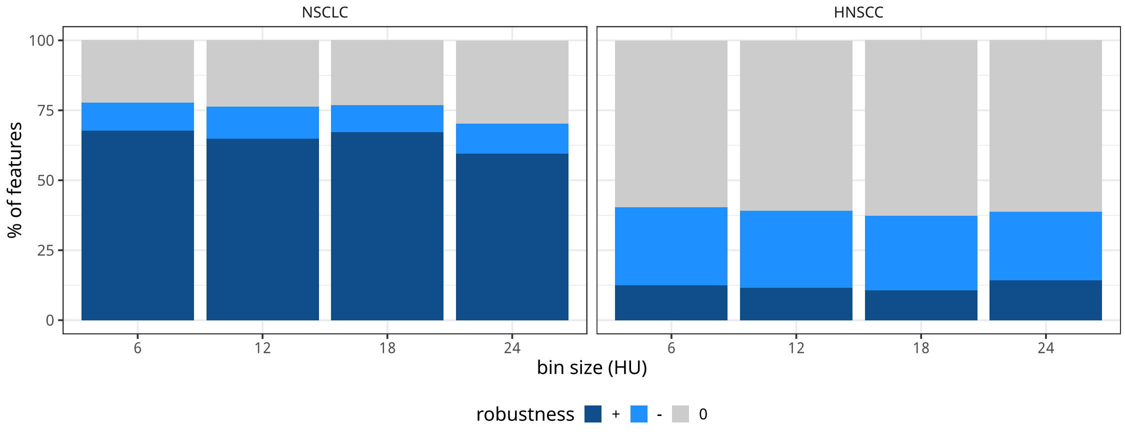

Figure S8 | Fraction of robust features in the test-retest set for different bin sizes used with fixed bin size discretisation. Robustness was determined using the 95% confidence interval (CI) of the intraclass correlation coefficient. Features with CI ≥ 0.90 were considered to be robust (+), CI < 0.90 non-robust (-), and indeterminate (0) otherwise.

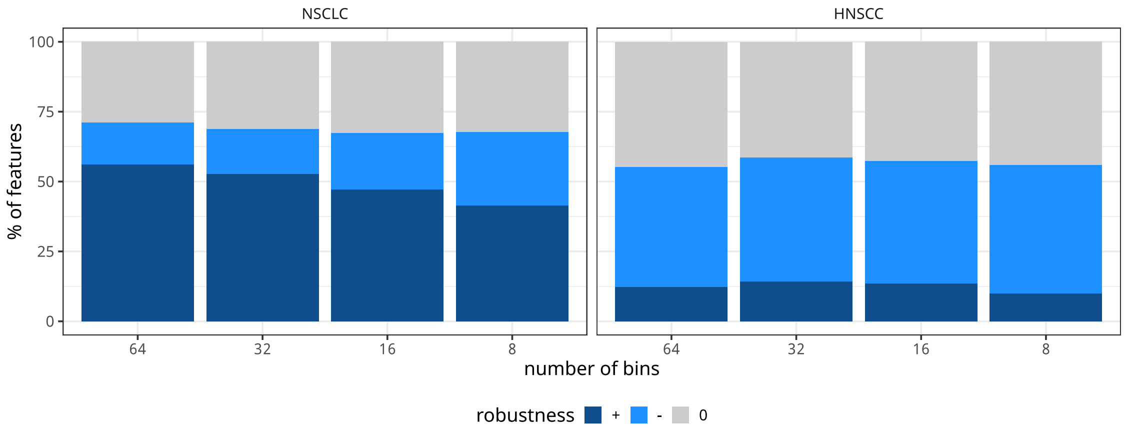

Figure S9 | Fraction of robust features in the test-retest set for different bin numbers used with fixed bin number discretisation. Robustness was determined using the 95% confidence interval (CI) of the intraclass correlation coefficient. Features with CI ≥ 0.90 were considered to be robust (+), CI < 0.90 non-robust (-), and indeterminate (0) otherwise.

Supplementary note 8: Robustness for different feature families

NSCLC and HNSCC cohorts have a different overall test-retest robustness. As some feature families contributed more features than others, we should assess whether the observed difference is caused by just a few large feature families. Test-retest robustness for features belonging to each family are summarised in Figure S10. With the exception of morphological features, the fraction of robust features in HNSCC was lower for all feature families.

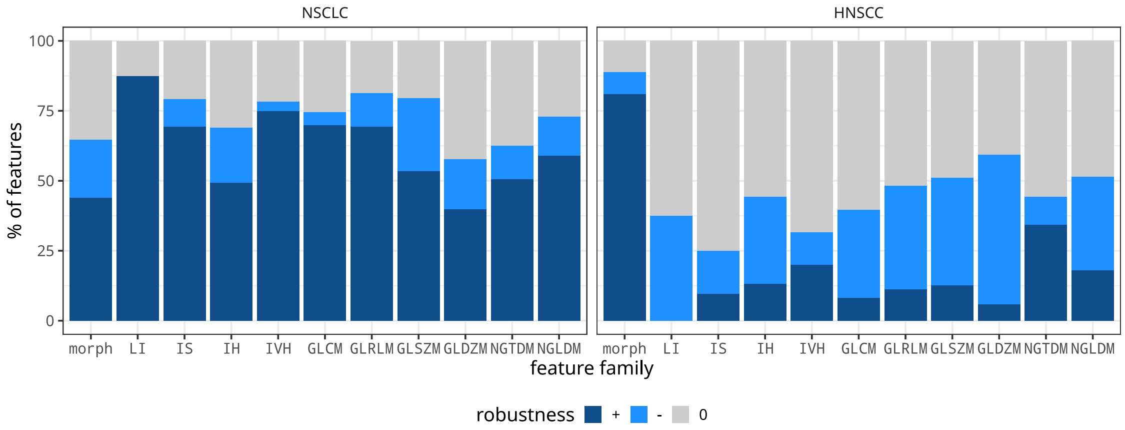

Figure S10 | Fraction of robust features identified in the test-retest set for different feature families. Robustness was assessed using the intraclass correlation coefficient (ICC). Features with ICC $\geq$ 0.90 were considered to be robust. morph: morphological features; LI: local intensity features; IS: intensity-based statistical features; IH: intensity-histogram features; IVH: intensity-volume histogram features; GLCM: grey level co-occurrence matrix-based texture features; GLRLM: grey level run length matrix-based texture features; GLSZM: grey level size zone matrix-based texture features; GLDZM: grey level distance zone matrix-based texture features; NGTDM: neighbourhood grey tone difference matrix-based features; and NGLDM: neighbouring grey level dependence matrix-based texture features.

References

[1] Chang, S. G., Yu, B. & Vetterli, M. Adaptive wavelet thresholding for image denoising and compression. *IEEE Transactions on Image Process.* **9**, 1532–1546 (2000). DOI 10.1109/83.862633.

[2] Mackin, D. *et al.* Effect of tube current on computed tomography radiomic features. *Sci. Reports* **8**, 2354 (2018). DOI 10.1038/s41598-018-20713-6.

[3] Derpanis, K. G. Fourier Transform of the Gaussian (2005). URL http://www.cse.yorku.ca/~kosta/CompVis_Notes/fourier_transform_Gaussian.pdf.

[4] Shrout, P. E. & Fleiss, J. L. Intraclass correlations: Uses in assessing rater reliability. *Psychol. Bull.* **86**, 420–428 (1979). DOI 10.1037/0033-2909.86.2.420.

[5] Zwanenburg, A., Leger, S., Vallières, M. & Löck, S. Image biomarker standardisation initiative. *eprint arXiv:1612.07003 [cs.CV]* (2016).

[6] Lorensen, W. E. & Cline, H. E. Marching cubes: A high resolution 3D surface construction algorithm. *ACM SIGGRAPH Comput. Graph.* **21**, 163–169 (1987). DOI 10.1145/37402.37422.

[7] Lewiner, T., Lopes, H., Vieira, A. W. & Tavares, G. Efficient Implementation of Marching Cubes' Cases with Topological Guarantees. *J. Graph. Tools* **8**, 1–15 (2003). DOI 10.1080/10867651.2003.10487582.

[8] Oliphant, T. E. Python for Scientific Computing. *Comput. Sci. & Eng.* **9**, 10–20 (2007). DOI 10.1109/MCSE.2007.58.

[9] Millman, K. J. & Aivazis, M. Python for Scientists and Engineers. *Comput. Sci. & Eng.* **13**, 9–12 (2011). DOI 10.1109/MCSE.2011.36.

[10] van der Walt, S. *et al.* scikit-image: image processing in Python. *PeerJ* **2**, e453 (2014). DOI 10.7717/peerj.453.

[11] Lee, G. *et al.* PyWavelets - Wavelet Transforms in Python (2006). URL https://github.com/PyWavelets/pywt.

[12] Ikeda, M., Makino, R., Imai, K., Matsumoto, M. & Hitomi, R. A method for estimating noise variance of CT image. *Comput. Med. Imaging Graph.* **34**, 642–650 (2010). DOI 10.1016/j.compmedimag.2010.07.005.

[13] Achanta, R. *et al.* SLIC superpixels compared to state-of-the-art superpixel methods. *IEEE transactions on pattern analysis machine intelligence* **34**, 2274–82 (2012). DOI 10.1109/TPAMI.2012.120.
